# Cannabidiol–Ion Channel Interactions Represent a Promising Preventive and Therapeutic Strategy in Hepatocellular Carcinoma

**DOI:** 10.3390/pathophysiology33010008

**Published:** 2026-01-14

**Authors:** María de Guadalupe Chávez-López, Arturo Avalos-Fuentes, Estrella del C. Cruz-Manzo, Pedro A. Aguirre-Arriaga, Benjamín Florán, Julio Isael Pérez-Carreón, Cecilia Bañuelos, Javier Camacho

**Affiliations:** 1 Departamento de Farmacología, Centro de Investigación y de Estudios Avanzados del Instituto Politécnico Nacional, Av. IPN 2508, Ciudad de Mexico 07360, Mexico; gchavez@cinvestav.mx (M.d.G.C.-L.); estrella.cruz@cinvestav.mx (E.d.C.C.-M.); antonio.aguirre@cinvestav.mx (P.A.A.-A.); 2 Departamento de Fisiología, Biofísica y Neurociencias, Centro de Investigación y de Estudios Avanzados del Instituto Politécnico Nacional, Av. IPN 2508, Ciudad de Mexico 07360, Mexico; javalos@cinvestav.mx (A.A.-F.); bfloran@fisio.cinvestav.mx (B.F.); 3 Instituto Nacional de Medicina Genómica (INMEGEN), Periférico Sur No. 4809, Col. Arenal Tepepan, Ciudad de Mexico 14610, Mexico; jiperez@inmegen.gob.mx; 4 Programa Transdisciplinario en Desarrollo Científico y Tecnológico para la Sociedad, Centro de Investigación y de Estudios Avanzados del Instituto Politécnico Nacional, Av. IPN 2508, Ciudad de Mexico 07360, Mexico; cebanuelos@cinvestav.mx

**Keywords:** cannabidiol, hepatocellular carcinoma, ion channels, drug repurposing, drug combination

## Abstract

Hepatocellular carcinoma (HCC) is the main type of liver cancer and one of the malignancies with the highest mortality rates worldwide. HCC is associated with diverse etiological factors including alcohol use, viral infections, fatty liver disease, and liver cirrhosis (a major risk factor for HCC). Unfortunately, many patients are diagnosed at advanced stages of the disease and receive palliative treatment only. Therefore, early markers of HCC and novel therapeutic approaches are urgently needed. The endocannabinoid system is involved in various physiological processes such as motor coordination, emotional control, learning and memory, neuronal development, antinociception, and immunological processes. Interestingly, endocannabinoids modulate signaling pathways involved in cell survival, proliferation, apoptosis, autophagy, and immune response. Consistently, several cannabinoids have demonstrated potential antitumor properties in experimental models. The participation of metabotropic and ionotropic cannabinoid receptors in the biological effects of cannabinoids has been extensively described. In addition, cannabinoids interact with other targets, including several ion channels. Notably, several ion channels targeted by cannabinoids are involved in inflammation, proliferation, and apoptosis in liver diseases, including HCC. In this literature review, we describe and discuss both the endocannabinoid system and exogenous phytocannabinoids, such as cannabidiol and Δ^9^-tetrahydrocannabinol, along with their canonical receptors, as well as the cannabidiol-targeted ion channels and their role in liver cancer and its preceding liver diseases. The cannabidiol-ion channel association is an extraordinary opportunity in liver cancer prevention and therapy, with potential implications for several environments that are for the benefit of cancer patients, including sociocultural, public health, and economic systems.

## 1. Introduction

Liver cancer remains a major global health challenge, ranking among the leading causes of cancer-related deaths [1]. Hepatocellular carcinoma (HCC), the predominant form of liver cancer, often emerges in the setting of cirrhosis, with both conditions representing advanced consequences of persistent liver injury and inflammation driven by hepatitis B or C virus infection, alcohol abuse, or the increasingly prevalent metabolic dysfunction-associated steatotic liver disease (MASLD) [2]. Notably, the global rise in MASLD has shifted the epidemiological landscape of HCC, positioning metabolic dysfunction as a growing contributor to liver cancer incidence in many regions [3]. Despite improvements in surveillance programs and the development of new therapeutic modalities, most patients are diagnosed at advanced stages, when curative options such as resection, transplantation, or local ablative therapies are no longer viable. In these cases, HCC treatment relies mainly on systemic approaches, including tyrosine kinase inhibitors and immune checkpoint blockades, which offer modest limited survival benefits and may be limited by resistance or treatment-related toxicity [4,5]. Hence, there is an urgent need for novel strategies for early detection, chemoprevention, and targeted therapy, including the discovery of reliable biomarkers for patient stratification and the design of mechanism-based interventions that are capable of preventing disease progression and improving long-term survival. In this context, phytochemical compounds have gained increasing attention as complementary or alternative anticancer agents, due to their capacity to modulate signaling pathways involved in tumor initiation, progression, and cell death [6]. Among them, cannabidiol (CBD), a non-psychoactive component of *Cannabis sativa*, has emerged as a promising molecule with potential preventive and therapeutic effects in various cancers, and an excellent recent review has summarized the role of CBD, primarily through CB1 and CB2 receptors, as potential therapeutic agent in liver cancer is available [7]. The purpose of this literature review is to describe and discuss several CBD molecular targets with special attention to cancer-associated ion channels and their role in liver cancer and its preceding liver diseases. We also discuss the possible clinical implications of these mechanisms and their potential relevance for innovation in liver cancer prevention and therapy. This review is also intended to encourage the study of the association between cannabidiol and ion channels and transporters as a therapeutic opportunity for drug combination and repurposing, as well as considering novel regulatory scenarios for the benefit of liver cancer patients. We reviewed the most updated information available (within five years) but also cite relevant previous publications.

## 2. The Endocannabinoid System

The endocannabinoid system (ECS) plays a crucial role in the development and functioning of several biological systems. Classically, the endocannabinoid system comprises receptors, endogenous ligands, and enzymes that synthesize, transport, and degrade such ligands. ECS regulates many biological processes, both in normal conditions like brain function, neurotransmitter release, sleep regulation, appetite, movement, and coordination, as well as pathological states such as neurodegenerative disorders, headaches, chronic pain, anxiety, depression, and cancer, among others. Accordingly, pharmacological modulation of the endocannabinoid system may be a potential target for preventing disease progression or enhancing symptom relief in multiple conditions, including cancer [8].

Most of the studies related to the endocannabinoid system typically focus on the CB1 and CB2 receptors, because they are widely expressed in biological systems. However, it is important to keep in mind that the endocannabinoid system also involves other GPCRs, such as GPR55, GPR119, and GPR18, as well as non-cannabinoid receptors, including serotonin, PPAR and adenosine receptors, and several ion channels, like TRPV [8].

### 2.1. Endocannabinoids

The most representative endocannabinoids are anandamide (AEA), which is isolated from the pig brain in 1992 and considered a partial agonist for cannabinoid receptors [9], and 2-arachidonoylglycerol (2-AG), which is isolated from the canine gut and considered a full agonist for cannabinoid receptors [10]. One key feature of endocannabinoids is that their precursors reside in membrane lipids. Due to its level of expression, 2-AG is considered the main endocannabinoid and the principal ligand for cannabinoid receptors in the CNS, as its levels have been reported to be up to 1000-fold higher than those of AEA [11]. These two endocannabinoids exhibit distinct pharmacological properties. AEA is a partial agonist for CB1 receptors and has almost no activity on CB2 receptors; on the other hand, 2-AG is a high-efficacy agonist for both CB1 and CB2 receptors [12]. Additionally, AEA and 2-AG can interact with other receptors. For example, AEA activates Transient Receptor Potential (TRP) channels, mainly TRPV1 channels, while 2-AG does not activate TRP channels [13]. Although 2-AG and AEA are the most studied endocannabinoids, the endocannabinoid family also includes virodhamin, noladine ether, N-arachidonoyldopamine (NADA), homo-linolenylethanolamide (HEA), docosatetraenylethanolamide (DEA), palmitoyletanolamide (PEA), and oleoylethanolamide (OEA) [14]. The synthesis of endocannabinoids is considered to be “on demand”, triggered by an increase in calcium levels in postsynaptic neurons [15].

### 2.2. Cannabinoid Receptors

In general, the effects of cannabinoids are mediated via G protein-coupled receptors (GPCRs), ion channels, and nuclear receptors. The principal GPCRs involved in the ECS are CB1 and CB2 receptors; however, other cannabinoid receptors have also been proposed, including GPR55, GPR18, and GPR119. Likewise, cannabinoids have been reported to activate TRP channels, such as TRPV1, TRPA1, TRPM1, TRPM8, and TRPML1 [16,17,18]. Finally, endocannabinoids, like anandamide, can activate nuclear receptors: specifically, PPARα and Pear [19].

#### CB1 and CB2 Receptors

Metabotropic cannabinoid receptors can be activated by three major groups of ligands:

1. Endocannabinoids, which are molecules formed endogenously by the body.
2. Phytocannabinoids, which are molecules formed by the cannabis plant.
3. Synthetic molecules or synthetic cannabinoids.

CB1 and CB2 receptors are members of the seven transmembrane domain receptors that are classically coupled to Gi/o proteins, and the result of their activation is the inhibition of the adenylyl cyclase pathway [20,21]. In addition, the βγ subunit can block N and P/Q voltage-gated calcium channels and activate GIRK channels [21]. The discovery of the main psychoactive component of the cannabis plant, Δ^9^-tetrahydrocannabinol (Δ^9^-THC), led to the production of synthetic cannabinoids and thus to the discovery and cloning of CB1 receptors from the rat cerebral cortex and one year later from human brain tissue [11,22,23]. CB1 receptors are composed of 472 amino acids in humans and 473 in rodents, with a similarity of 97–99% across species. CB1 receptor expression is widely distributed in organs and tissues, including the pancreas, testes, adipocytes, T lymphocytes, lung, liver, and peripheral nervous system, and is primarily in the central nervous system. The CB1 receptor is among the most abundant GPCR in the CNS [24]. On the other hand, the CB2 receptor consists of 360 amino acids in humans. It was cloned from human promyelocytic leukemia line HL-60 cells three years after the discovery of the CB1 receptors. The CB2 receptor shares only 44% of amino acid sequence homology with the CB1 receptor [11,25]. The CB2 receptor, unlike the CB1 receptor, was considered a peripheral receptor due to its high expression in immune cells and organs such as the spleen [25]. Subsequently, expression of CB2 receptors in the CNS was found in perivascular glia [26]. Recently, a greater presence of CB2 receptors has been reported in CNS neurons, in areas such as the prefrontal cortex, hippocampus, thalamus, striatum, olfactory bulb, amygdala, VTA, SN pars compacta and pars reticulata, and the cerebellum. In these areas, CB2 receptors are found both pre- and postsynaptically, which modulates neuronal excitability and neurotransmitter release [27,28,29].

### 2.3. Endocannabinoid Synthesis and Degradation

Endocannabinoids are synthesized from membrane-derived phospholipid precursors and released “on demand”. In the central nervous system, they primarily function as retrograde messengers; that is, they are synthesized at the postsynaptic level and activate their targets at the presynapse [15].

#### 2.3.1. Anandamide (AEA)

N-arachidonylethanolamine, also known as anandamide, was the first endocannabinoid isolated by Devane et al. in 1992 from a pig brain [9]. The synthesis of AEA is relatively short; it is synthesized from glycerophospholipid N-acyl-phosphatidylethanolamine (NAPE) and catalyzed by NAPE-phospholipase D (NAPE-PLD) [9,11,27,30,31]. Although this is the primary route of AEA synthesis, an alternative route involving PLA2 has also been reported [32]. Although the mechanism of AEA diffusion is not fully understood, it is thought to cross membranes by passive diffusion to reach its target receptor [31]. Another theory is that AEA is stored in lipid droplets (adiposomes), which act as reservoirs, and it is transported to the plasma membrane [33]. Finally, AEA is degraded into arachidonic acid and ethanolamine, mostly by fatty acid amide hydrolase (FAAH) [31,33].

#### 2.3.2. 2-Arachidonoylglycerol (2-AG)

Discovered by Mechoulam et al. [10], 2-AG is synthesized from PIP2 by PLC-β generating diacylglycerol (DAG), and finally, through the action of both DAG lipases (DAGLα and DAGLβ), it is converted into 2-AG. Unlike AEA, 2-AG is synthesized primarily in postsynaptic neurons and is the most abundant endocannabinoid in the CNS [32]. It is known that its synthesis is “on demand” because it depends on increases in the intracellular calcium concentration [11,34]. As with AEA, the transport of 2-AG into and out of the cell is unclear; however, there is experimental evidence suggesting that 2-AG and AEA can be transported across the membrane by Fatty Acid Binding Protein 5 (FABP5), which may facilitate their intracellular trafficking [35]. Finally, 2-AG is predominantly hydrolyzed by monoacylglycerol lipase (MAGL) into arachidonic acid and glycerol [34].

## 3. Phytocannabinoids

For centuries, numerous beneficial effects have been attributed to marijuana use, including emotional and physical benefits, among others. Phytocannabinoids refer to bioactive terpenoid molecules that are primarily isolated from the *Cannabis sativa* plant [36]. The phytocannabinoid biosynthesis in *Cannabis sativa* has been well elucidated; however, other phytocannabinoid sources have recently emerged [37]. *Cannabis sativa* has been shown to contain approximately 120 phytocannabinoids, which have been classified into several chemical classes. The phytocannabinoids Δ^9^-tetrahydrocannabinol (Δ^9^-THC) and cannabidiol (CBD) are the most studied extracts from *Cannabis sativa*, with Δ^9^-THC being the most abundant, comprising approximately 17.3% of the total cannabinoid content, and CBD comprising 7.7% [38].

### 3.1. Δ^9^-Tetrahydrocannabinol (Δ^9^-THC)

Δ^9^-THC was discovered in 1964 by Gaioni and Mechoulam. It is the principal psychoactive component of *Cannabis sativa* [39]. For this reason, clinical use is currently minimal, except in chemotherapy as an antiemetic and, in some cases, for managing symptoms of autoimmune diseases [36]. Interestingly, the discovery of a membrane receptor that bound Δ^9^-THC led to efforts to identify the ECS. It is now known that Δ^9^-THC psychotropic effects are due to its affinity for and binding to CB1 receptors [40]. Despite Δ^9^-THC’s high affinity for CB1 receptors, it is considered a partial agonist, as demonstrated by GTPγS binding experiments [38,41]. Evidence also suggests that Δ^9^-THC binds to CB2 receptors, albeit with lower affinity than to CB1 receptors [41]. Moreover, it has been observed that Δ^9^-THC behaves as a CB2 receptor antagonist [42]. In this sense, Δ^9^-THC can also activate putative cannabinoid receptors such as GPR55 and GPR18 [43]. Interestingly, it has been proposed that Δ^9^-THC can activate other receptors that do not properly belong to the ECS. One of them is the 5-HT3A receptor, where the presence of Δ^9^-THC drastically decreases channel currents, likely via a mechanism involving the regulation of an allosteric site [44]. Another receptor reported to be a target of Δ^9^-THC is the glycine receptor, which increases the amplitude of Gly currents in neurons of the rat ventral tegmental area, enhancing their activity through an allosteric mechanism [45]. The PPARγ can also be activated by Δ^9^-THC, increasing superoxide dismutase activity and thereby increasing hydrogen peroxide (H_2_O_2_), with a dose-dependent vasorelaxant effect on the aorta and superior mesenteric arteries [46].

### 3.2. Cannabidiol (CBD)

CBD was isolated by Mechoulam and Shvo in 1963, one year before Δ^9^-THC [47]. Unlike Δ^9^-THC, which induces psychoactive effects, CBD is considered to be a major non-psychoactive phytocannabinoid in cannabis, with reported antioxidant and anti-inflammatory properties, as well as antidepressant, anxiolytic, anticonvulsant, antinausea, antirheumatoid, antipain (in cancer mainly), and antipsychotic properties [37,48,49,50]. Recent studies suggest that CBD may exert anti-cancer effects or help to control symptoms related to the disease. CBD induces apoptosis in HL-60 myeloblastic leukemia cells and glioma cells [48,51,52], and has emerged as a promising therapeutic agent for human hepatocellular carcinoma [7]. CBD is currently considered the third most abundant phytocannabinoid, having a presence of up to 7.7% of the total phytocannabinoid content in cannabis and second in pharmacological relevance after Δ^9^-THC [38]. CBD is a lipophilic molecule, is quickly absorbed, and easily crosses biological barriers [53]; therefore, its distribution throughout the body is wide. In this regard, it is important to mention that CBD administration has a favorable safety profile in terms of administration doses and side effects [54].

## 4. Cannabidiol Pharmacological Targets

The pharmacological targets of CBD include not only receptors belonging to the ECS, but can also various non-cannabinoid receptors, enzymes, transporters, and cellular uptake proteins [36].

### 4.1. CBD and Cannabinoid Receptors

CBD can primarily exert its pharmacological effects in the body through binding to the most expressed cannabinoid CB1 and CB2 receptors, but it has also been shown to bind to GPR55 and GPR18. Unlike most cannabinoids and endocannabinoids, CBD has a low affinity for CB1 and CB2 receptors. Although CBD has a low affinity for the CB1 receptor, it can negatively modulate its activity, acting as a negative allosteric modulator, decreasing the efficacy of 2-AG and Δ^9^-THC, which are ligands that easily bind to CB1 receptors [55]. Consistent with this mechanism, the administration of CBD decreases the psychotic symptoms generated by Δ^9^-THC [56]. As with CB1 receptors, CBD can bind to CB2 receptors, functioning as an antagonist or inverse agonist [49,57]. Interestingly, although CBD directly regulates the CB1 and CB2 receptors, it also inhibits the metabolism of AEA, thereby modulating the endocannabinoid tone [50,52]. There are other putative cannabinoid receptors: GPR55, GPR18, and the lesser studied GPR3, GPR6, and GPR12. GPR55 and GPR18 receptors can be bound by CBD, acting as an antagonist for both receptors [43,50,58]. On the other hand, CBD acts as an inverse agonist for GPR3, GPR6, and GPR12 [59].

Several studies have shown that both CB1 and CB2 are expressed in many cancer types. However, receptor stimulation can lead to different outcomes, with protective effects on some tumor subtypes and unfavorable effects on others [60,61]. Overexpression of CB1 and CB2 receptors is correlated with an improved prognosis in HCC [62]. Interestingly, the activation of CB2 promotes tumor cell death and growth suppression. Additionally, CBD has been demonstrated to promote apoptosis in several gastric cancer cell lines [63]. Furthermore, some studies suggest that GPR18 may influence the migration and proliferation of cancer cells, macrophages, and other inflammation-associated immune cells. Thus, they may be potential targets for inflammation, cancer, and analgesia therapy [64]. GPR55 is overexpressed in several types of cancer, such as colon, pancreatic, breast, prostate, and ovarian, and has been linked to cell proliferation, migration, and metastasis [65,66,67]. A recent work demonstrated that CBD reverses the malignant phenotype of HCC cells via the GPR55/TP53/MAPK axis [68]. Finally, it is suggested that GPR12 may be a potential CBD target for preventing metastasis [69].

### 4.2. CBD and Non-Cannabinoid Receptors

#### 4.2.1. Serotonin Receptors (5-HT)

Serotonin receptors have already been implicated in the therapeutic effect of CBD. CBD, although it has a modest affinity, acts as an agonist for 5-HT1A receptors [50,70]. In behavioral models in rats, CBD improves stress-induced anxiety symptoms by activating 5-HT1A receptors, in a similar way to buspirone [71]. Additionally, a neuroprotective effect of CBD has been associated with 5-HT1A receptors in a model of middle cerebral artery (MCA) occlusion [72]. Regarding 5-HT2A receptors, CBD acts as a partial agonist or antagonist, exhibiting a lower affinity for 5-HT2A than for 5-HT1A [70], and is considered a non-competitive antagonist of 5-HT3 receptors [73].

Interestingly, serotonin promotes cancer progression by promoting proliferation via cell cycle progression, autophagy, and the suppression of apoptosis. HCC cells express different serotonin receptors, like 1A, 1B, 2B, and 7. Notably, serum and platelet-driven serotonin levels are higher in patients with HCC compared to individuals without cancer. In addition, serum serotonin levels are upregulated in a chemical-induced HCC mouse model, suggesting its involvement in the prognosis and progression of HCC [74].

#### 4.2.2. Adenosine Receptors

Adenosine receptors are GPCRs that are widely expressed in the body and activated by adenosine. A1 and A3 are receptors that couple to Gi/o proteins, while A2A and A2B receptors couple to Gs proteins. The therapeutic effects of CBD on adenosine receptors primarily focus on the activation of A1 and A2A receptors; this is considered a positive allosteric modulator (PAM) [59]. CBD suppresses ischemia-induced ventricular arrhythmia through A1 receptor activation [75]. One of the most notable and widely reported effects of CBD on A2A receptors is its anti-inflammatory action. This effect is primarily mediated by the reduction in proinflammatory cytokines [76]. In addition, CBD improves adenosine signaling, possibly by inhibiting adenosine reuptake, and thus reducing inflammatory processes [77]. Therefore, CBD can be used in pathologies that involve inflammatory processes such as rheumatoid arthritis and multiple sclerosis [49,78].

Adenosine, through its receptors A1, A2A, A2B, and A3, can potently suppress the function of T and NK cells [79,80]. Moreover, there is evidence that the A2B receptor plays a crucial role in tumor cell proliferation, angiogenesis, metastasis, and immune suppression [81]. Notably, the purinergic signaling axis is a mechanism of tumor-mediated immunosuppression that has garnered attention as a potential therapeutic target.

#### 4.2.3. Peroxisome Proliferator-Activated Receptor Gamma (PPARγ)

PPARγ is a nuclear receptor that regulates the expression of genes involved in lipid metabolism, glucose homeostasis, inflammation, and cell differentiation. Normally, the activation of PPAR receptors has an anti-inflammatory effect, and CBD acts as an activator of PPARs. Several reports have shown that CBD exerts a protective effect through PPAR activation; for example, it protects the BBB (blood–brain barrier) from damage in an ischemic stroke model (OGD) via the activation of PPAR [82]. Also, several reports implicate the beneficial effect of PPAR activation by CBD in Alzheimer’s disease (AD). In this sense, CBD promotes neuronal survival by decreasing the apoptotic rate in an AD model through PPAR activation [83]. Additionally, CBD reduces AB-induced neuroinflammation and increases hippocampal neurogenesis via PPAR activation [84]. Regarding multiple sclerosis, CBD may contribute to a therapeutic effect by reducing proinflammatory cytokines through the upregulation and activation of PPARγ receptors [85]. Interestingly, in vitro PPARγ activation has been associated with growth inhibition, cellular differentiation, and changes in the cell cycle in various solid tumors, including prostate, gastric, and lung adenocarcinoma, as well as esophageal, renal, and breast cancer [86]. However, one study found that targeting PPARγ counteracts tumor adaptation to immune checkpoint blockade in HCC [87].

## 5. Ion Channels and Transporters: Their Role in Liver Cancer

CBD targets other very relevant proteins in health and disease: namely, ion channels. Ion channels are membrane proteins that allow for ion flux and can be activated by different stimuli, including changes in membrane potential, temperature or pH, different types of ligands, and mechanical force [88]. The role of ion channels in maintaining cellular homeostasis is of the utmost importance and impacts phenomena such as neurotransmission, hormone release, cardiac function, and sensory processes, among others. Therefore, ion channel dysregulation is associated with major pathological conditions [88]. The association between carcinogenesis and ion channel and transporter activity/expression is a major field that has gained great attention because of its potential use for cancer diagnosis, treatment, and prognosis [89,90]. Because several excellent reviews have covered this topic [89,90,91,92], here we will focus on those channels and transporters associated with liver cancer and its preceding diseases.

The liver produces and secretes various compounds that serve to break down and emulsify fats, in addition to breaking down red blood cells. It also synthesizes hormones, regulates glycogen storage, and eliminates toxins and drugs from the body. Consequently, hepatocytes are equipped with a wide variety of ion channels/transporters, many of which are dysregulated in HCC [92].

To date, a large number of dysregulated channels and transporters have been identified in liver cancer. This abnormality primarily contributes to the proliferation and evasion of apoptosis in cancer cells, involving various signaling pathways. Numerous studies have shown that blocking certain dysregulated ion channels in liver cancer contributes to decreased cell proliferation, migration, invasion, and the promotion of apoptosis in several models [92,93,94,95,96]. Some dysregulated channels and transporters in liver cancer, along with the affected cellular processes or potential roles, are listed in Table 1.

One of the major issues to solve is to unravel the precise molecular mechanism of action associating ion channels and transporters with liver cancer. The participation of ion channels in health and disease is not restricted to their roles as ion-conducting proteins in the plasma membrane. Many ion channels also bind to other proteins and locate in organelles including mitochondria, endoplasmic reticulum, lysosomes, and the nucleus [89,90,91]. Thus, despite the fact that some channels and transporters are dysregulated in cancer in opposite directions, the precise mechanism of action of the contribution of ion channels and transporters in cancer may depend on several factors, including their location and association with other proteins. For instance, most of the potassium channels dysregulated in liver cancer are upregulated, but others are downregulated, and the same situation has been observed for some aquaporins (Table 1). Is this opposite regulation a cellular compensating response to regulate the role and expression of ion channels and transporters? Or does every channel and transporter have very specific roles in cancer? On the other hand, the cellular mechanisms associating ion channels and cancer may not be related to the cell membrane potential in all cases. For example, overexpression of potassium channels in liver cancer may hyperpolarize the membrane, while overexpression of sodium channels may depolarize it. More research on the molecular mechanisms of action of the participation of ion channels and transporters in cancer is warranted. Anyway, this knowledge holds great potential for developing new therapeutic strategies, such as drug combinations targeting a dysregulated channel or transporter, or molecules in the related signaling pathways. For instance, ABC transporters associated with multidrug resistance are overexpressed (Table 1); thus, it is feasible to develop drug combinations targeting one of these transporters in liver cancer and avoid anticancer drug efflux.

**Table 1:** Ion channels and transporters dysregulated in liver cancer.

Channel/ Transporter	Transported Ion	Expression Change	Cellular Process or Mechanism Involved	References
K_Ca_3.1	K^+^	Overexpression	Cell proliferation, invasion, metastasis	[97,98,99]
K_v_7.1	K^+^	Downregulation	Tumor suppressor, prognosis	[100]
K_ir_6.2	K^+^	Overexpression	Tumor progression	[101]
K_v_10.1	K^+^	Overexpression	Cell proliferation	[94,102,103]
Ca_v_3.1	Ca^2+^	Overexpression	Cell proliferation	[104]
Ca_v_3.2	Ca^2+^	Overexpression	Cell proliferation	[104]
Ca_v_3.3	Ca^2+^	Overexpression	Cell proliferation	[104]
P2X3	Na^+^, Ca^2+^	Overexpression	Cell proliferation, prognosis	[105]
NCX1	Na^+^/Ca^2+^	Overexpression	Migration, invasion	[106]
CIC-3	Cl^−^	Overexpression	Cell cycle, tumor size, prognosis	[107,108]
CIC-4	Cl^−^	Downregulation	Cell proliferation, invasion, survival, migration	[109]
CLIC1	Cl^−^	Overexpression	Prognosis, migration, invasion	[110,111,112,113]
CLIC2	Cl^−^	Downregulation	Tight junction protein expression	[113,114]
CLIC5	Cl^−^	Overexpression	Migration, invasion	[113,115]
GABA_A_R	Cl^−^	Downregulation	Migration, invasion, metastasis, tumor growth	[116,117,118]
NHE1	Na^+^/H^1+^	Overexpression	Clinical stage, invasion, migration, survival, apoptosis inhibition	[119,120,121,122]
VGSCβ1	Na^+^	Downregulation	Migration, invasion	[123]
Na_v_1.2	Na^+^	Overexpression	Prognosis	[124,125]
ASIC1a	Na^+^	Overexpression	Migration, invasion, cell proliferation	[126,127]
AQP5	Water channel	Overexpression	Metastasis	[128,129]
AQP9	Water channel	Downregulation	Prognosis, invasion, migration, cell proliferation	[129,130]
TRPC6	Ca^2+^	Overexpression	Migration, invasion	[106,131,132]
TRPC1	Non-selective cation	Overexpression	Prognosis, cell proliferation	[133]
TRPV1	Non-selective cation	Downregulation	Prognosis, Ca^2+^ homeostasis, apoptosis	[134,135,136]
TRPV2	Non-selective cation	Overexpression	Prognosis, cytotoxicity, cancer stemness	[137]
TRPV4	Non-selective cation	Overexpression	Cell proliferation, metastasis, biomarkers	[138,139,140]
TRPM7	Ca^2+^, Mg^2+^	Overexpression	Cell proliferation, metastasis	[141,142]
TRPM8		Overexpression	Mitochondrial function, prognosis	[143,144]
ITPR3	Ca^2+^	Overexpression	Prognosis, apoptosis	[145,146]
α7nAChR	Ca^2+^	Overexpression	Chemoresistance, tumorigenesis	[147,148]
OCT1 OCT3	Organic cation transporter	Downregulation	Prognosis, response to TKI’s, cell proliferation	[149,150,151]
ABCB1 ABCC1 ABCC2 ABCC3	Organic anion transporters	Overexpression	Multidrug resistance	[152,153]
ABCC5	Organic anion transporters	Overexpression	Biomarker	[154]
MCT1	Monocarboxylate transporter	Overexpression	Metastasis, glycolysis, L-Lactate transport	[155,156]
MCT4	Monocarboxylate transporter	Overexpression	Prognosis, cell proliferation, migration, L-Lactate transport	[156,157]
VDAC1	Anion channel	Overexpression	Autophagy	[158]

## 6. Ion Channels as Targets for CBD

As mentioned before, CBD has generated enormous interest among the scientific community due to its wide variety of effects on different conditions such as pain, inflammation, epilepsy, anxiety, and even cancer, among others [159,160,161,162,163]. Therefore, many studies have been dedicated to identifying the potential targets and mechanisms of CBD in different conditions to harness its therapeutic potential. It is well known that CBD is a multi-target molecule, and in addition to CB1 and CB2 receptors, it interacts with approximately 65 molecular targets [164] where it can behave as an activator or inhibitor, including a variety of ion channels involved in liver cancer. Table 2 lists the ion channels targeted by CBD, indicating their dysregulation in liver cancer where available.

**Table 2:** CBD activity in ion channels and their dysregulation in liver cancer.

Ion Channel	CBD Activity	Dysregulation in Liver Cancer	Reference
Ca_v_3.1 Ca_v_3.2 Ca_v_3.3	Inhibitor	Overexpression	[59,104,165,166,167]
Na_v_1.1 Na_v_1.3–1.7	Inhibitor	Not reported	[59,166,167,168]
Na_v_1.2	Inhibitor	Overexpression	[59,124,125,166,167]
VDAC1	Inhibitor	Overexpression	[59,158,169]
K_v_2.1	Inhibitor	Not reported	[59]
K_v_7.1	Inhibitor	Downregulation	[100,166,167]
K_v_11.1	Inhibitor	Not reported	[166,167]
TRPV1	Activator	Downregulation	[18,59,134,135,136,166]
TRPV2	Activator	Overexpression	[18,59,137,166]
TRPV3	Activator	Not reported	[18,59,166]
TRPV4	Activator	Overexpression	[18,59,138,139,140,166]
TRPA1	Activator	Not reported	[18,59,166]
TRPM8	Inhibitor	Overexpression	[18,59,143,144,166]
ABCB1 (Transporter)	Inhibitor	Overexpression	[7,152,153,170]
GlyRs	Activator	Not reported	[59,166]
GABA_A_R	Activator	Downregulation (subunit α6, β3 y ε)	[59,116,117,118,166]
5HT3A	Inhibitor	Not reported	[59,166]
α7-nAChR	Inhibitor	Overexpression	[59,147,148,166]

The diversity of ion channels targeted by CBD represents a plethora of opportunities to regulate several cellular processes that are relevant to liver cancer. Calcium regulates multiple cellular processes such as excitability, muscle contraction, neurotransmitter release, mitosis, and apoptosis, in addition to serving as a second messenger in various intracellular signaling pathways [167,171]. Several studies have shown that CBD acts as a Ca_v_3 channel family inhibitor, causing various effects like modifying cell excitability or inhibiting Ca^2+^-induced Ca^2+^ release, etc. [59,166,167,172]. Several Ca_v_ channels are differentially expressed in cancer cells compared to normal cells and contribute to proliferation, cell-cycle progression, and apoptosis [173]. Interestingly, CBD elevates cytosolic-free Ca^2+^ in leukemia and gives rise to cytotoxicity [165]. Furthermore, overexpression of Ca_v_3.1, Ca_v_3.2, and Ca_v_3.3 channels has been found in liver cancer, and it has been shown that these channels participate in hepatocellular carcinoma cell proliferation [104].

Dysregulation of voltage-gated sodium channels causes the development of several diseases. CBD is a non-selective Na_v_1.1–1.7 sodium channel inhibitor and is effective in the treatment of epilepsy [59,167,168,174]. Some Na_v_ channels are aberrantly expressed in cancer tissues such as breast, lung, prostate, colon, and cervix. Their dysregulated expression has been associated with metastatic progression and cancer-related death [175]. Notably, Na_v_1.2 channel overexpression has been reported in liver cancer, which has been associated with better survival of HCC patients [124,125]. Thus, the role of ion channel overexpression on cancer progression may be channel-specific.

Voltage-dependent anion channel 1 (VDAC1) is located in the outer mitochondrial membrane and is involved in controlling cellular energetics, metabolic homeostasis, and apoptosis by mediating the transfer of metabolites between mitochondria and cytosol. CBD inhibits the VDAC1 channel; this inhibition may be responsible for the immunosuppressive and anticancer effects of CBD [59,169]. A recent study on metformin’s anticancer mechanism used a label-free drug affinity responsive target stability (DARTS)-LC-MS/MS method and found that the VDAC1 channel is a novel binding protein involved in autophagy-related cell death induction by high-dose metformin in HCC [158].

Potassium channel dysregulation in cancer promotes cell proliferation, apoptosis resistance, metabolic changes, angiogenesis, and metastasis, emerging as targets for development of new therapeutic drugs [176]. A 2020 study showed that CBD suppresses K_v_7.1 and K_v_11.1 channels, which underlie the slow (IKs) and rapid (IKr) components of the cardiac delayed-rectifier current, respectively [166,167,177]. Interestingly, KCNQ1 (K_v_7.1) has been reported to be downregulated in HCC and may suppress metastasis in this disease [100].

The TRP channel family is expressed in a wide variety of tissues and is responsible for monitoring mechanical, thermal, and chemical stimuli. The vanilloid (TRPV), canonical (TRPC), melastatin (TRPM), and ankyrin (TRPA) channels have gained great interest for their role in proliferation, migration, and invasion, among other functions in different cancers [178]. CBD activates TRPV1, TRPV2, TRPV3, TRPV4, and TRPA1, and antagonizes TRPM8. Some of CBD’s anti-hyperalgesia and anti-inflammatory properties can be explained by interactions with these channels [18,166]. Regarding liver cancer, downregulation of TRPV1 is associated with poor prognosis; accordingly, TRPV1 activation disturbs Ca^2+^ homeostasis, leading to apoptosis in liver cancer cell lines [134,135,136]. Additionally, TRPV2 overexpression has been found in moderate and well differentiated liver cancer compared to poorly differentiated liver cancer; as expected, channel activation inhibits tumor growth in vivo and mediates H_2_O_2_-induced oxidative stress and cell death in liver cancer cell lines [137]. The high TRPV4 expression in liver cancer has been reported in some patients and the TRPV4 blockade suppresses proliferation and metastasis. Interestingly, patients with a low expression profile of TRPV4 and MCOLN3 are more likely to benefit from immunotherapy [138,139,140]. TRPM8 is widely expressed in different tissues, but mainly in the prostate, and its dysregulation has been found in several types of cancer, such as melanoma, breast, and prostate. Notably, recent studies have determined an overexpression of TRPM8 in liver cancer; in addition, in vivo and in vitro studies showed that inhibition of the channel suppresses hepatocarcinogenesis [143,144].

The development of multidrug resistance (MDR) is the main cause of chemotherapy failure, which is generally due to the decreased intracellular drug concentration that is usually associated with ABC family transporter overexpression, such as ABCB1 (P-glycoprotein, MDR1) [152]. Very interestingly, it has been found that CBD can inhibit ABCB1 function, increasing the intracellular concentration of chemotherapeutics. This finding may imply a possible MDR reversal mechanism by CBD [7,170], since ABCB1 is overexpressed in liver cancer, mainly in chemotherapeutic resistant cells [152,153].

Gamma-aminobutyric acid (GABA) plays a key role in the nervous system by facilitating inhibitory neurotransmission, leading to a reduction in neuronal activity through interaction with GABA_A_ receptors. CBD is able to increase GABA_A_R-mediated currents in a dose-dependent manner, with the β subunit being its preferential binding site [59,166]. The role of GABA and its receptors in cancer is controversial because it has a variety of effects depending on tissue type. In liver cancer, the α6, β3, and ε subunits are downregulated, and their activation produces suppression of migration and invasion, decreased tumor growth in vivo, and attenuation of tumor-initiating stem cell (TISC) proliferation [116,117,118].

Five classes of nicotinic nAchR receptor subunits (α, β, γ, ε, and δ) have been described and the α7-nAChR is widely expressed in the nervous system. Excitatory and inhibitory synaptic transmission can be modulated by these receptors and reduction in their expression has been associated with a predisposition to seizures. CBD can inhibit the α7-nAChR in a dose-dependent manner [59]. In addition, α7-nAChR is associated with diverse pathologies, including cancer, where it is overexpressed. Interestingly, in liver cancer, a relationship has been demonstrated between the development and progression of this malignancy and the activation of the α7-nAChR; in addition, it favors chemoresistance to sorafenib [147,148].

**Figure 1:**
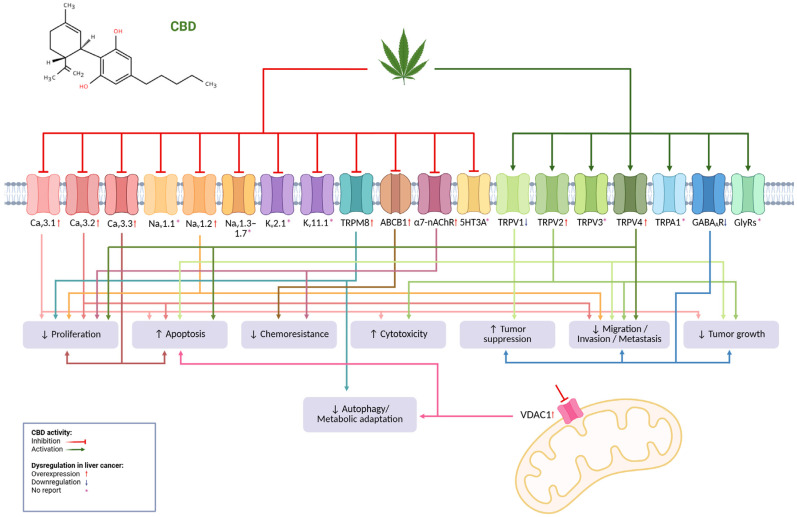
Schematic CDB targeting and effect on ion channels dysregulated in liver cancer. CBD activates some ion channels while inhibiting other channels. Dysregulation of ion channels in liver cancer results in either overexpression or downregulation of the channels. Figure created in www.biorender.com (accessed on 27 October 2025).

Targeting dysregulated ion channels in liver cancer with CBD represents an extraordinary opportunity to propose novel anticancer treatments. CBD exhibits a high safety profile and is also a multi-target molecule that is capable of inhibiting and activating a significant number of dysregulated ion channels in liver cancer (Figure 1). Therefore, it is sensible to consider CBD—for instance, in combination with other drugs—to fight liver cancer.

Unfortunately, in many cases, liver cancer is detected at late stages, where patients receive palliative treatment only. Thus, it is very important to find earlier potential interventions, avoiding the occurrence of late stages. Notably, several experimental approaches using different models of liver diseases strongly suggest CBD as a promising preventive agent. Next, we will discuss these interesting hepato-protective effects of CBD on liver diseases that predispose people to liver cancer.

## 7. CBD Effects on Liver Diseases and Liver Cancer: Preventive and Therapeutic Opportunities

As mentioned, CBD is one of the major phytocannabinoids derived from *Cannabis sativa*, which is widely studied for its pharmacological profile and lack of psychotropic effects and, more recently, for its potential role in liver pathophysiology [179]. Various liver diseases share common mechanisms of injury, including chronic inflammation, oxidative stress, and activation of hepatic stellate cells. Given the rising global prevalence of liver diseases and the limited therapeutic options that are currently available, the exploration of CBD as a hepatoprotective and antineoplastic agent is gaining translational relevance. In this context, CBD has been proposed as a potential modulator of liver processes, with potential implications for preventing liver damage and modulating tumor-related processes.

Numerous preclinical studies have explored the effects of CBD on different experimental models of liver injury, demonstrating protective and restorative actions on liver function, and these are summarized in Table 3. In hepatotoxicity models, CBD reduces oxidative stress, inflammation, and uncontrolled apoptosis, preserving the liver architecture and function against toxic or metabolic insults. In models of steatosis and fibrosis, CBD regulates key pathways such as NF-κB, NLRP3, PPAR-α, and AMPK, limiting progression toward cirrhosis and improving metabolic parameters.

Beyond its preventive potential, several preclinical studies demonstrate that cannabinoids, particularly CBD, are effective in experimental models of hepatocellular carcinoma (Table 3). In vitro and in vivo HCC models reveal that CBD directly suppresses tumor cell proliferation, migration, invasion, and metastatic dissemination, while promoting programmed cell death through apoptotic and pyroptotic mechanisms. These antitumor effects have been mechanistically linked to the activation of tumor suppressors and stress-response pathways, including p53 and CHOP/ATF4, as well as the inhibition of pro-oncogenic signaling cascades such as GPR55/MAPK and the tumor glycolytic metabolism. These multifactorial actions support its potential as an adjuvant or sensitizing agent in combination cancer therapies, as observed with doxorubicin and cabozantinib [170,180]. Collectively, these data indicate that CBD not only may prevent the progression of chronic liver injury toward cirrhosis and HCC but that it also protects the liver against hepatocarcinoma development and counteracts cancer propagation. The plethora of liver processes regulated by cannabinoids offers several options to create a hypothesis and develop translational research: for instance, in drug-combination or drug-repurposing approaches.

**Table 3:** Effects of CBD on liver diseases (preclinical models).

Model/Disease	Cell Lines/Animal Models	CBD Concentration/ Dose	Effect(s)	Possible Molecular Mechanism(s) Involved	Potential Clinical Implications	Reference
Cadmium (CdCl_2_)-induced acute hepatotoxicity	Male Sprague-Dawley rats	5 mg/kg	CBD inhibited hepatic lipid peroxidation, significantly decreased serum ALT and MDA levels, restored GSH and NO, and improved hepatic architecture, showing reduced necrosis and vascular congestion compared with the Cd-treated group only.	CBD reduces oxidative stress (↓ MDA, ↑ GSH, ↑ NO, ↑ catalase) and suppresses the activation of inflammatory and apoptotic pathways (↓ TNF-α, ↓ COX-2, ↓ NF-κB, ↓ caspase-3, ↓ caspase-9).	Preventive/therapeutic: CBD protects the liver against heavy metal-induced toxicity (cadmium) through antioxidant, anti-inflammatory, and antiapoptotic effects, suggesting clinical potential in chemical hepatotoxicity prevention.	[181]
Cocaine-induced acute hepatotoxicity	Male Swiss mice	30 mg/kg	CBD decreased serum ALT levels and indocyanine green (ICG) retention, reduced hepatic necrosis and inflammation, preserved tissue architecture, and prevented cocaine-induced seizures and lethality.	CBD exerts anti-inflammatory and antioxidant effects by modulating immune responses, reducing leukocyte infiltration, and attenuating hepatic inflammatory injury.	Preventive/therapeutic: CBD exhibits hepatoprotective properties against cocaine-induced acute toxicity, with potential applicability in toxic and drug-induced liver injury.	[182]
Concanavalin A-induced acute hepatitis	C57BL/6 mice	5–50 mg/kg	CBD reduced serum AST levels, hepatic necrosis, and mononuclear infiltration, while increasing hepatic MDSC accumulation.	CBD activates TRPV1 receptors and induces the expansion of myeloid-derived suppressor cells (MDSCs, CD11b^+^Gr-1^+^) with high arginase expression, thereby suppressing T-cell proliferation and hepatic inflammatory responses.	Preventive/therapeutic: CBD attenuates acute liver inflammation and injury by modulating immune responses through TRPV1 activation, showing therapeutic potential in autoimmune or toxic hepatitis.	[183]
Acute alcohol-induced hepatic steatosis	HepG2 E47 cells (expressing CYP2E1) C57BL/6 mice	5 µM 5 mg/kg	CBD reduced ethanol-induced hepatic injury and steatosis; decreased serum AST and triglyceride levels; prevented ATP depletion; reduced ROS and oxidative markers; and increased autophagy in hepatocytes.	CBD reduces oxidative stress (↓ ROS, ↓ 4-HNE), inhibits the JNK/MAPK pathway, and increases autophagy levels (↑ LC3-II/LC3-I).	Preventive/therapeutic: CBD protects against acute alcohol-induced liver injury by reducing oxidative stress and restoring autophagy, thereby preventing progression to steatohepatitis, fibrosis, or hepatocellular carcinoma.	[184]
Oleic acid-induced hepatic steatosis (MASLD)	HHL-5 and 3T3-L1 cells. Female ob/ob mice, zebrafish (*Danio rerio*) embryos and larvae	5 y 10 μM 3 mg/kg	CBD reduced intracellular triglyceride levels in hepatocytes and adipocytes in a dose-dependent manner, increased GSH, ATP, and NAD levels (indicating enhanced mitochondrial activity), and decreased hepatic lipid accumulation in ob/ob mice and zebrafish models.	CBD increases phosphorylation of key regulators of energy metabolism (↑ AMPKα2, ↑ ERK1/2, ↑ STAT2/3/6, ↑ CREB, ↑ PRAS40), promoting lipolysis, mitochondrial β-oxidation, and reduced lipogenesis. This effect is independent of CB1 and TRPV1 receptors.	Preventive/therapeutic: CBD improves lipid metabolism and mitochondrial function, reducing hepatic steatosis. It acts as a potential preventive and therapeutic agent for MASLD, which is capable of reversing early metabolic damage associated with obesity and metabolic syndrome.	[185]
Chronic alcohol-induced hepatic steatosis	C57BL/6 J mice	5 and 10 mg/kg	CBD reduced inflammation, oxidative/nitrosative stress, and hepatic lipid accumulation, improving liver structure and function in the alcohol-induced injury model.	CBD attenuates inflammatory pathway activation (↓ TNF-α, ↓ MCP1, ↓ IL-1β, ↓ MIP2, ↓ E-selectin), reduces oxidative/nitrosative stress (↓ NOX2, ↓ 3-nitrotyrosine), and modulates lipid metabolism (↓ FASN, ↓ ACC1; ↑ PPARα, ↑ CPT-1, ↑ ADIPOR1, ↑ MCAD).	Preventive/therapeutic: CBD protects against alcohol-induced liver injury and may prevent progression toward fibrosis or hepatocellular carcinoma.	[186]
Non-alcoholic steatohepatitis induced by high-fat, high-cholesterol diet (MASLD)	RAW264.7 cells. Male C57BL/6 J mice	5 μM 5 mg/kg	CBD decreased serum ALT, hepatic lipids, and proinflammatory cytokines (IL-1β, TNF-α, MCP-1); reduced macrophage infiltration (CD68^+^); and improved hepatic architecture, attenuating diet-induced steatohepatitis.	CBD inhibits NF-κB/NLRP3 activation (↓ p-IκBα, ↓ p-NF-κBp65, ↓ caspase-1p20, ↓ IL-1β), leading to reduced hepatic inflammation and oxidative stress.	Preventive/therapeutic: CBD attenuates inflammation and liver injury induced by a high-fat and high-cholesterol diet by modulating the NF-κB/NLRP3 pathway, showing therapeutic potential in MASLD.	[187]
Alcohol and high-fat, high-cholesterol diet-induced liver injury (EHFD)	Male C57BL/6 J mice	5 mg/kg	CBD attenuated hepatic steatosis and injury (↓ TG, ↓ hepatic cholesterol, ↓ serum ALT/AST), reduced oxidative stress (improved GSH/GSSG ratio, ↓ MDA), decreased macrophage infiltration (↓ CD68), downregulated proinflammatory cytokines (↓ IL-1β, ↓ MCP-1, ↓ TNF-α), and inhibited the NF-κB/NLRP3/pyroptosis axis.	CBD inhibits NF-κB activation, thereby reducing NLRP3 inflammasome initiation signaling; it also suppresses NLRP3 inflammasome activation by decreasing caspase-1 expression and GSDMD cleavage, leading to reduced pyroptosis and hepatic inflammation.	Therapeutic: CBD protects against liver injury induced by a combination of alcohol and high-fat diet through modulation of inflammation and programmed cell death (pyroptosis), suggesting potential clinical value in alcoholic liver disease associated with metabolic disorders.	[188]
Metabolic syndrome-associated hepatic steatosis induced by a high-fat, high-cholesterol diet (MASLD)	Male C57BL/6 J mice	2.39 mg/kg	CBD attenuated systemic and hepatic inflammation and had a partial effect on intestinal dysbiosis; however, its impact on hepatic steatosis was limited.	CBD reduced hepatic expression of inflammatory markers (↓ TNF-α, ↓ iNOS), partially modulated gut microbiota composition, and improved glucose tolerance.	Preventive/therapeutic: CBD shows potential to modulate inflammation and metabolic dysfunction in the context of metabolic syndrome and MASLD.	[189]
CCl_4_-induced hepatic fibrosis	Male C57BL/6 J mice	20 mg/kg	CBD exhibited strong anti-inflammatory and antifibrotic activities, reducing hepatic fibrosis, fibroblast migration, and associated inflammation.	CBD reduces TGF-β and IL-4-induced fibroblast migration, exerting anti-inflammatory and antifibrotic effects that limit hepatic injury progression.	Preventive/therapeutic: CBD shows potential to prevent or attenuate toxin-induced hepatic fibrosis by modulating inflammation and fibroblast activation, which may lower the risk of progression to cirrhosis or hepatocellular carcinoma.	[190]
CCl_4_-induced hepatic fibrosis	Male C57BL/6 J mice	4 mg/kg y 8 mg/kg	CBD improved liver function (↓ AST, ↓ HA), decreased histological damage and collagen deposition, attenuated fibrosis (↓ α-SMA, ↓ COL-I), and reduced inflammatory infiltration and cytokine levels (IL-6, TNF-α, IL-1β).	CBD inhibits NF-κB activation (↓ p-NF-κB, ↓ p-IκBα, ↓ COX-2) and activates PPAR-α; it also reduces p38 MAPK signaling.	Preventive/therapeutic: CBD exerts hepatoprotective and antifibrotic effects by modulating the NF-κB and PPAR-α pathways, suggesting potential use in preventing progression toward cirrhosis or hepatocellular carcinoma.	[191]
PFOS-induced liver injury and fibrosis	RAW264.7, AML12, and LX-2 cells Male C57BL/6 J mice	10 μM 10 mg/kg	CBD decreased TNF-α, IL-1β, IL-6, α-SMA, and collagen I expression; lowered serum AST, ALT, and LDH levels; and improved hepatic architecture and mitochondrial integrity.	CBD inhibits macrophage extracellular trap (MET) formation by binding to PAD4, thereby downregulating the CCDC25–ILK–NF-κB axis and reducing inflammation and hepatic stellate cell activation.	Preventive/therapeutic: CBD protects against PFOS-induced inflammation and fibrosis by modulating the PAD4–MET–CCDC25–ILK–NF-κB axis. Its antifibrotic and anti-inflammatory effects suggest potential to prevent HCC progression associated with chronic environmental contaminant exposure.	[192]
Liver fibrosis	HSCs cells	5 µM	CBD induced rapid, selective death of activated HSCs, without affecting healthy hepatocytes or quiescent HSCs; it activated ER stress and JNK pathways, thereby eliminating the cells responsible for fibrosis with high specificity.	CBD induces endoplasmic reticulum (ER) stress, (↑ PERK, ↑ ATF6, ↑ IRE1), which triggers the IRE1–ASK1–JNK pathway and results in apoptosis. This effect is independent of cannabinoid receptors (CB1/CB2).	Preventive/therapeutic: By selectively eliminating activated HSCs, CBD may slow or reverse hepatic fibrosis—a central mechanism to prevent progression to cirrhosis and, eventually, hepatocellular carcinoma.	[193]
Viral hepatitis (HBV and HCV)	HepG2 2.2.15 cells (HBV model), Huh7.5 cells (HCV model)	10 µM	CBD significantly suppressed HCV replication without affecting cell viability or HBV replication.	CBD inhibits HCV replication (↓ viral RNA ~85%) with minimal cytotoxicity (<3%). It did not affect HBV replication, suggesting a possible indirect immunomodulatory effect mediated by CB2 receptors.	Preventive/therapeutic: CBD may attenuate inflammation and hepatic disease progression associated with viral hepatitis, thereby reducing the risk of fibrosis or HCC development through inhibition of HCV replication.	[194]
Diethylnitrosamine (DENA)-induced hepatocellular carcinoma	Male Wistar rats	3–30 mg/kg	CBD reduced serum levels of ALT, AST, GGT, and AFP, improved hepatic architecture, and decreased tumor aggressiveness in DENA-treated rats.	CBD inhibits the Hedgehog signaling pathway (↓ Smo, ↓ Ptch-1, ↓ Gli-1, ↓ Hhip); this restores redox balance (↑ SOD, ↑ CAT, ↓ MDA), promoting apoptosis.	Preventive/therapeutic: CBD exhibits a dual role by attenuating the progression of hepatic injury toward HCC and exerting direct antitumor effects via modulation of the Hedgehog pathway and oxidative stress.	[195]
Hepatocellular carcinoma	HepG2 cells	1 µM y 5 µM	CBD significantly decreased exosome and microvesicles release.	CBD reduces the expression of vesicular and mitochondrial markers (CD63, p-STAT3, prohibitin), suggesting mitochondrial modulation.	Therapeutic: CBD acts as a modulator of tumor intercellular communication by interfering with extracellular vesicle release, which could attenuate tumor progression and therapeutic resistance in HCC.	[196]
Hepatocellular carcinoma	HepG2, Huh7, MHCC97H, and HCCLM3 cells Female athymic nude mice	40 µM 40 mg/kg	CBD suppressed HCC cell growth both in vitro and in vivo, induced caspase-3/GSDME-dependent pyroptosis, and repressed aerobic glycolysis.	CBD activates the integrated stress response (ISR) and mitochondrial stress, leading to the upregulation of ATF4, CHOP, and IGFBP1. This cascade activates caspase-3 and GSDME, promoting pyroptosis. Additionally, IGFBP1 inhibits the AKT/GSK3β axis, thereby reducing glycolysis.	Therapeutic: CBD acts as a potential direct antitumor agent by inducing pyroptosis and blocking tumor glycolysis, which could enhance therapeutic efficacy in HCC.	[197]
Hepatocellular carcinoma	Huh-7 and SNU398 cells. Female BALB/c nude mice	100 nM 10 mg/kg	CBD reduced proliferation, migration, invasion, and metastasis of HCC cells; it also enhanced apoptosis and decreased tumor growth and hepatic metastatic nodule formation.	CBD downregulates GPR55 expression, leading to increased TP53 levels, inhibits the MAPK pathway (↓ p-JNK, ↓ p-p38, ↓ p-MEK1/2), induces apoptosis, and suppresses the epithelial–mesenchymal transition (EMT) (↓ N-cadherin, ↑ E-cadherin).	Therapeutic: CBD reverses the malignant phenotype of HCC by modulating the GPR55/TP53/MAPK axis, demonstrating strong antitumor and antimetastatic potential.	[68]
Co-treatment with doxorubicin in hepatocellular carcinoma	BNL1 ME cells	10 µM	CBD increased the sensitivity of HCC cells to doxorubicin; the combination reduced cell viability and proliferation, allowing the use of lower DOX concentration and demonstrating a synergistic effect with a potential reduction in treatment-associated toxicity.	CBD activates TRPV2 channels, promoting doxorubicin (DOX) uptake, inhibits P-gp (P-glycoprotein ATPase), and increases intracellular drug accumulation, thereby enhancing apoptosis.	Therapeutic: The combination of CBD with doxorubicin enhances antitumor efficacy and may allow for a reduction in the required chemotherapeutic dose.	[170]
Co-treatment with cabozantinib in hepatocellular carcinoma	HepG2 and Hep3B cells	1–100 µM	CBD increased the sensitivity of HCC cells to cabozantinib, leading to a significant increase in apoptosis and a reduction in cell viability.	CBD induces endoplasmic reticulum (ER) stress, activates phosphorylated p53, and promotes apoptosis independently of cannabinoid receptors (CNR1/CNR2).	Therapeutic: The combination of CBD with cabozantinib enhances antitumor efficacy, potentially allowing dose reduction and improving therapeutic response.	[180]

Interestingly, the preclinical findings of the preventive or therapeutic effect of CBD on liver diseases (described in Table 3) are in accordance with epidemiological observations. Population-based studies including thousands of clinical records have reported a lower prevalence or slower progression of liver disease and HCC (reductions of 38–55%) among cannabis users compared to non-users [198,199]. While these studies did not evaluate CBD in isolation, their findings support the hypothesis that certain cannabinoids may help as preventive or therapeutic agents in liver diseases [200].

Notably, several clinical trials are exploring the use of CBD in liver and oncological disorders, primarily for its anti-inflammatory and antifibrotic effects, as well as its potential to improve tolerance to conventional treatments. A summary of these clinical studies is presented in Table 4. In a Phase 2 trial evaluating GWP42003 (CBD) in patients with fatty liver disease (Clinical trial NCT01284634), changes in hepatic fat content were assessed by using magnetic resonance-based techniques; although the study confirmed an acceptable safety profile, no conclusive reductions in liver steatosis were observed. In contrast, a Phase I study in patients with biochemically recurrent prostate cancer treated with Epidiolex (CBD, Clinical trial NCT04428203) demonstrated good tolerability at doses up to 800 mg/day, with most patients exhibiting biochemical disease stability and a subset of patients showing partial reductions in prostate-specific antigen (PSA) levels. Although this study was not designed to assess antitumor efficacy, it provides preliminary clinical signals of the biological activity of CBD in an oncological context.

Although not all the trials listed have published results yet, the completed and ongoing studies suggest that the potential clinical use of CBD is not restricted to liver tissue but extends to other malignancies such as prostate and other cancers. Likewise, trials in conditions like Parkinson’s disease and COVID-19 provide supportive evidence for the systemic safety and immunomodulatory properties of CBD. We included broader clinical trials to exemplify the translational potential of CBD beyond liver cancer and propose the biological plausibility that similar mechanisms could be therapeutically exploited in HCC: for instance, in combination with immunotherapy.

**Table 4:** Overview of clinical trials for cannabidiol investigation in liver diseases, liver cancer, and cancer. Key search terms included the following: “Cannabidiol/CBD+, liver cancer, cancer, hepatocellular carcinoma, liver disease”. (www.clinicaltrials.gov, accessed 30 October 2025).

Trial Name	Conditions	Phase	Status	NCT
Study to Evaluate the Effect of GWP42003 (CBD) on Liver Fat Levels in Participants with Fatty Liver Disease	Fatty liver	II	Completed	NCT01284634
Cannabidiol for Reducing Drinking in Alcohol Use Disorder (CARAMEL)	Alcohol Use Disorder	II	Recruiting	NCT05159830
A Phase 2a Study to Evaluate the Safety and Efficacy of Cannabidiol Only as Maintenance Therapy and Steroid Sparing in Patients with Stable Autoimmune Hepatitis	Autoimmune Hepatitis	II	Terminated	NCT04129489
Outcomes Mandate National Integration with Cannabis as Medicine (OMNI-Can)	Chronic Pain, Chronic Pain Syndrome, Chronic Pain Due to Injury, Chronic Pain Due to Trauma, Fibromyalgia, Seizures Hepatitis C, Cancer Crohn Disease, HIV/AIDS, Multiple Sclerosis, Traumatic Brain Injury, Sickle Cell Disease, Post-Traumatic Stress Disorder, Tourette Syndrome, Ulcerative Colitis, Glaucoma, Epilepsy, Inflammatory Bowel Diseases, Parkinson Disease, Amyotrophic Lateral Sclerosis, Chronic Traumatic Encephalopathy, Anxiety, Depression Insomnia, Autism, Opioid-Use Disorder, Bipolar Disorder, SARS-CoV Infection, COVID-19, Corona Virus Infection, Coronavirus	II	Recruiting	NCT03944447
A Pilot Study on the Effect of Cannabis Oil in Untreatable Liver Cancer Patients (CanHep)	Hepatocellular Carcinoma	II	Recruiting	NCT06518434
A Study of the Efficacy of Cannabidiol in Patients with Multiple Myeloma, Glioblastoma Multiforme, and GI Malignancies	Cancer of Pancreas, Cancer of Liver, Cancer of Rectum, Cancer of Colon, Cancer, Gall Bladder, Myeloma Multiple, Glioblastoma Multiforme	I II	Unknown status	NCT03607643
A Study: Pure CBD as Single-Agent for Solid Tumor.	Solid Tumor	II	Unknown status	NCT02255292
CBD for Breast Cancer Primary Tumors	Breast Cancer	I	Not yet recruiting	NCT06148038
Epidiolex (CBD) in Patients with Biochemically Recurrent Prostate Cancer	Prostate Cancer, Recurrent Prostate Cancer, Prostate Adenocarcinoma	I	Completed	NCT04428203
Investigating the Potential Role of a Novel Quadrate Combination Therapy Mifepristone (Antiprogestrone), Tamoxifen, Retinoic Acid and Cannabidiol (Selective Cyp 26 Inhibitor) for Treating Early Breast Cancer.	Female Breast Cancer	III	Unknow status	NCT05016349

Taken together, preclinical, epidemiological, and clinical data converge to support CBD as a promising candidate for the prevention and management of liver diseases and HCC, with potential implications for sociocultural, public health, and economic systems.

## 8. Prospective Translational, Regulatory, and Social Implications

The increasing use of cannabis and cannabinoids with different purposes makes it imperative to standardize the indications for the benefit of people and public health systems [201,202]. The potential use of CBD in liver diseases entails an evolving regulatory landscape for cannabidiol-based interventions. A more precise understanding of how CBD modulates the ion channels implicated in hepatocarcinogenesis can generate the mechanistic evidence that regulatory agencies require to refine benefit–risk assessments, establish exposure limits, and guide safety monitoring for cannabinoid-derived products. In the long term, integrating data on efficacy, toxicity, and off-target effects at the ion-channel level may support clinical trial authorization. Importantly, this mechanistic framework also opens up the possibility of developing combination therapeutic strategies involving CBD, to enhance efficacy or mitigate toxicity through ion-channel-related mechanisms.

At the population level, identifying ion-channel-related biomarkers may provide tools for early detection, precision medicine, and surveillance in patients with chronic liver disease. In this way, a solid pathophysiological framework describing CBD–ion channel interactions may facilitate the implementation of public health strategies to reduce the burden of liver cancer.

**Figure 2:**
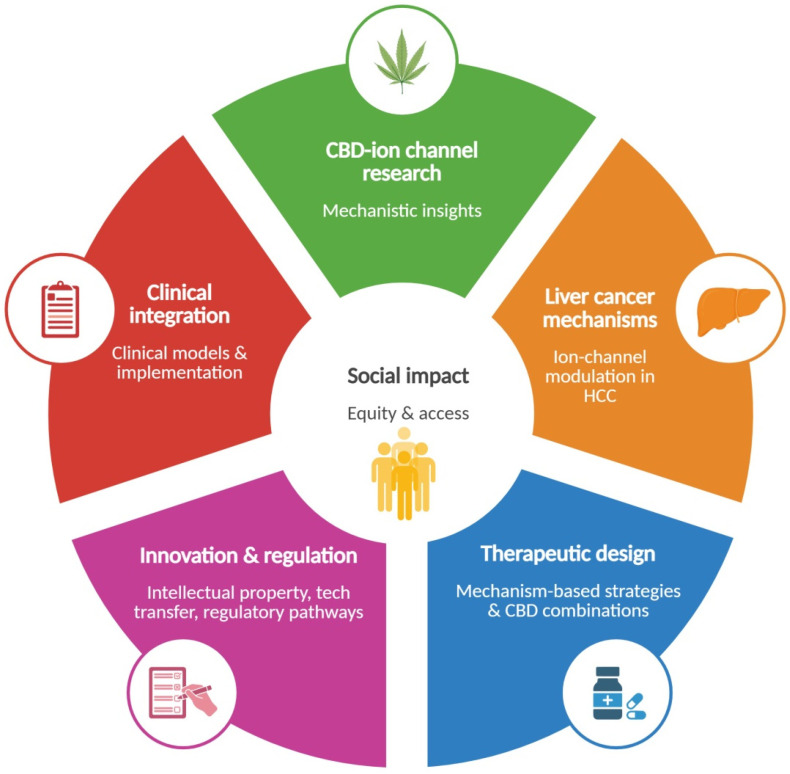
Prospective translational, regulatory, and social implications of CBD use in liver diseases. The bench-to-bedside pathway on CBD–ion channel research in liver diseases should drive to the development of mechanism-based therapeutic strategies, including CBD-based drug combinations. This approach requires innovation and adherence to regulatory standards. These advances ultimately connect to clinical implementation and to broader social impact through improved equity and access to emerging treatments. Figure created in www.biorender.com (accessed on 27 October 2025).

Beyond regulatory and clinical considerations, these developments also entail relevant social implications. Emerging evidence on cannabinoid uses in diverse medical settings including oncology [203,204] coexists with heterogeneous public perceptions and, in some contexts, with stigma or misinformation. This highlights the need for health-literacy strategies that help distinguish clinically evaluated therapeutics from unregulated cannabis-derived products. Moreover, the development of CBD-based therapies may narrow the current health inequities associated with high costs, geographic availability, or lack of coverage within healthcare systems. This approach may guide policies that ensure equitable access. Finally, the therapeutic potential of ion-channel-targeted CBD interventions create opportunities for intellectual property development, technology-transfer processes, and new lines of translational research. Figure 2 summarizes the potential impact of CBD use in liver diseases in several environments.

## 9. Conclusions

Forthcoming research should include drug combinations: for instance, CBD and immunotherapy or CBD and novel tyrosine kinase inhibitors. Population studies identifying lower incidence of liver diseases or cancer in patients by using specific drugs which may be combined with CBD will also be very helpful. These approaches may facilitate the development of clinical trials with the advantages of drug repurposing. Of course, in vivo validation of specific ion channel–CBD interactions is also warranted. Exploiting the cannabidiol-ion channel-transporters association represents an extraordinary opportunity for liver cancer prevention and therapy, which may help to reduce the high mortality from this malignancy and to involve sociocultural, public health, regulatory, and economic systems.

## Data Availability

Not applicable.

## Abbreviations

4-HNE	4-Hydroxynonenal
ACC1	Acetyl-CoA carboxylase 1
ADIPOR1	Adiponectin receptor 1
AFP	Alpha-fetoprotein
ALT	Alanine aminotransferase
AMPKα2	AMP-activated protein kinase alpha 2 subunit
ASK1	Apoptosis signal-regulating kinase 1
AST	Aspartate aminotransferase
ATF4	Activating transcription factor 4
ATF6	Activating transcription factor 6
CAT	Catalase
CB1/CB2	Cannabinoid receptors type 1 and type 2
CCDC25	Coiled-coil domain containing 25
CD63	Tetraspanin associated with exosomes (vesicular marker CD63)
CHOP	C/EBP homologous protein (ER stress marker)
CNR1/CNR2	Genes encoding cannabinoid receptors CB1 and CB2
COL-I	Collagen type I
COX-2	Cyclooxygenase-2
CPT-1	Carnitine palmitoyltransferase 1
CREB	cAMP response element-binding protein
EHFD	Ethanol and high-fat diet
EMT	Epithelial–mesenchymal transition
ERK1/2	Extracellular signal-regulated kinases 1 and 2
FASN	Fatty acid synthase
GGT	Gamma-glutamyltransferase
Gli-1	GLI family zinc finger 1 (Hedgehog pathway transcription factor)
GPR55	G protein-coupled receptor 55
GSDMD	Gasdermin D
GSDME	Gasdermin E
GSK3β	Glycogen synthase kinase 3 beta
GSSG	Oxidized glutathione
HA	Hyaluronic acid
HCC	Hepatocellular carcinoma
Hhip	Hedgehog-interacting protein
IGFBP1	Insulin-like growth factor binding protein 1
ILK	Integrin-linked kinase
iNOS	Inducible nitric oxide synthase
IRE1	Inositol-requiring enzyme 1
IκBα	Inhibitor of kappa B alpha
JNK	c-Jun N-terminal kinase
LC3-II/LC3-I	Microtubule-associated protein 1A/1B-light chain 3 (autophagy marker)
LDH	Lactate dehydrogenase
MASLD	Metabolic dysfunction-associated steatotic liver disease
MCAD	Medium-chain acyl-CoA dehydrogenase
MCP1	Monocyte chemoattractant protein-1
MDA	Malondialdehyde
MIP2	Macrophage inflammatory protein 2
NAD	Nicotinamide adenine dinucleotide
NF-κB	Nuclear factor kappa B
NLRP3	NOD-like receptor family, pyrin domain-containing 3
NO	Nitric oxide
NOX2	NADPH oxidase 2
p38	Mitogen-activated protein kinase p38
PAD4	Peptidyl arginine deiminase 4
PERK	Protein kinase RNA-like endoplasmic reticulum kinase
p-MEK1/2	Phosphorylated mitogen-activated protein kinase 1/2
p-NF-κBp65	Phosphorylated NF-κB p65 subunit
PPAR-α	Peroxisome proliferator-activated receptor alpha
PRAS40	Proline-rich Akt substrate of 40 kDa
p-STAT3	Phosphorylated signal transducer and activator of transcription 3
Ptch-1	Patched-1 (Hedgehog pathway receptor)
ROS	Reactive oxygen species
Smo	Smoothened (Hedgehog pathway protein)
SOD	Superoxide dismutase
TG	Triglycerides
TGF-β	Transforming growth factor beta
TNF-α	Tumor necrosis factor Alpha
TP53	Tumor suppressor gene p53
TRPV1	Transient receptor potential vanilloid type 1
TRPV2	Transient receptor potential vanilloid type 2
α-SMA	Alpha-smooth muscle actin
